# A review of cell death pathways in hemorrhagic stroke

**DOI:** 10.3389/fcell.2025.1570569

**Published:** 2025-04-28

**Authors:** John H. Rinald, Carol M. Troy

**Affiliations:** ^1^ Neurobiology and Behavior PhD Program, Columbia University, New York, NY, United States; ^2^ Department of Pathology and Cell Biology, Vagelos College of Physicians and Surgeons, Columbia University, New York, NY, United States; ^3^ Department of Neurology, Vagelos College of Physicians and Surgeons, Columbia University, New York, NY, United States; ^4^ The Taub Institute for Research on Alzheimer's Disease and the Aging Brain, Vagelos College of Physicians and Surgeons, Columbia University, New York, NY, United States

**Keywords:** hemorrhagic stroke, cell death, apoptosis, inflammation, neurovascular disease, caspases

## Abstract

Hemorrhagic stroke is a debilitating neurological disease, affecting millions worldwide. Characterized by bleeding in the brain, it is caused by a breakdown of the blood-brain barrier (BBB) and causes damage through the presence of iron in the brain, immune activation and increased intracranial pressure. The goal of this mini-review is to explore the signaling pathways that lead to cell death that are a part of disease progression in hemorrhagic stroke. This mini-review will highlight clinical observations and data, while also incorporating findings using preclinical disease models. There are important roles for apoptosis, necroptosis, necrosis, autophagy, ferroptosis, and pyroptosis in hemorrhagic stroke. Recent work has highlighted the interplay between these phenomena, providing key regulators as potential therapeutic targets, including reactive oxygen species, iron metabolism, and caspases. Therapeutic strategies that can delay or counteract the cytotoxic effects of hemorrhage can improve clinical outcomes in hemorrhagic stroke patients.

## 1 Introduction

Stroke is the third leading cause of death and disability globally (Feigin et al., 2022). There are two categories of stroke: ischemic, in which blood flow through blood vessels is occluded, and hemorrhagic, in which a blood vessel ruptures, and blood spills into the neural parenchyma (Kuriakose and Xiao, 2020). Hemorrhagic stroke can be further classified by the location of the hemorrhage into subarachnoid hemorrhage, and intracerebral hemorrhage. Subarachnoid hemorrhage and intracerebral hemorrhage have very different clinical presentations, and different treatment options (Coelho et al., 2016; An et al., 2017). Subarachnoid hemorrhage typically presents as a sudden onset severe headache. Intracerebral hemorrhage manifests differently depending on the affected brain region, e.g., thalamic bleeds typically cause unilateral motor impacts. However, while the clinical manifestations of these diseases are different, the cellular and molecular pathology is comparable. In this mini-review, we will be discussing the cellular effects of hemorrhagic stroke, specifically exploring the pathological mechanisms of cell death that are involved in hemorrhagic stroke.

Hemorrhagic stroke significantly impacts neurological function by destroying areas of the brain and preventing the normal function of neural behavioral circuits. The pathology of hemorrhagic stroke changes as the disease progresses past the initial time of injury (Magid-Bernstein et al., 2022). Primary injury occurs within the first 2 days, and is largely mediated by the mass effect of the hematoma in the neural parenchyma (Zazulia et al., 1999). Inside the hemorrhagic core, there is a high concentration of toxic blood components due to the lysis of hemoglobin-filled erythrocytes (Fang et al., 2022). This can cause neuronal death through, for example, an increase in reactive oxygen species (ROS) due to increased local iron concentrations (Koduri et al., 2022). Additionally, the increase of intracranial pressure from the mass of the blood can cause cell death through restricting flow of blood to other regions of the brain (Zazulia et al., 1999). Secondary injury occurs over a longer time period, and is largely understood to be the consequences of several toxic pathways, including: inflammation, excitotoxicity, oxidative stress, and hemolysis (Ziai, 2013; Shoamanesh and Katsanos, 2021). These pathways can actually be observed within the first hour after stroke, but continue to have deleterious effects even after hematoma clearance (Bautista et al., 2021). Both the primary and secondary injuries cause irreversible neuronal death, which causes the neurological effects of stroke.

**FIGURE 1:**
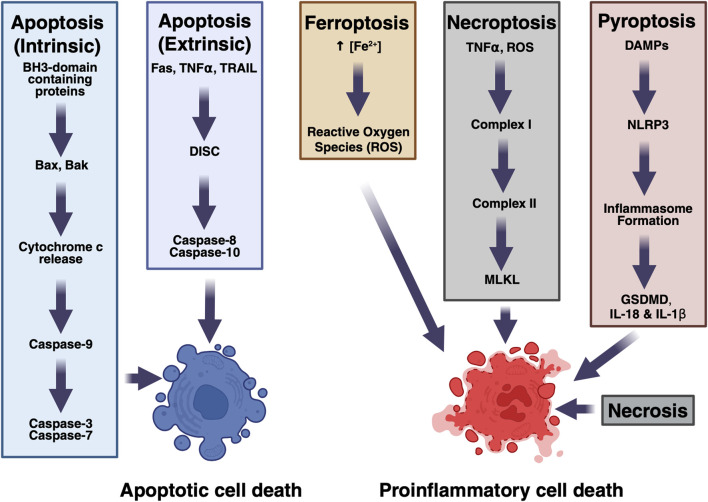
Mechanisms of cell death in hemorrhagic stroke. Multiple pathways contribute to cell death.

In hemorrhagic stroke, there are multiple modes of cell death active at any time, depicted in Figure 1. For example, in the regions closest to the injury, tissue is quickly liquified via necrosis, whereas in the peri-hematoma regions the majority of the dying cells appear apoptotic, rather than necrotic at several timepoints after injury (Qureshi et al., 2003). This initial finding that both apoptosis and necrosis play roles in hemorrhagic stroke has expanded to the discoveries that many other types of cell death are also present in hemorrhagic stroke (Zhang et al., 2022). By studying the plurality of cell death modes in hemorrhagic stroke, we can better understand the molecular pathways that are responsible for the devastating consequences of this disease and develop more specific disease-modifying strategies.

## 2 Apoptosis

Apoptosis was the first active form of cell death discovered. Apoptosis requires energy, and is pathologically characterized by cell shrinkage, chromatin condensation and the formation of apoptotic bodies (Coleman et al., 2001). Although apoptosis is often called programmed cell death, programmed cell death is normal death by apoptosis that occurs during development and maintenance of homeostasis; apoptosis is also responsible for cell death during disease (Elmore, 2007).

Depending on the stimulation, cells can either undergo intrinsic or extrinsic apoptosis (Green, 2000). These pathways have been well described previously (Qureshi et al., 2003; Green, 2000; Xu and Shi, 2007; Moujalled et al., 2021). Briefly, intrinsic apoptosis, also known as the mitochondrial apoptotic pathway, can be a response to various intracellular stressors, including DNA damage, hypoxia, or oxidative stress (Westphal et al., 2014). These pathways activate p53, leading to the expression of BH3-domain containing proteins including Bim and Bid, which bind to and activate Bax and Bak. Bax and Bak form pores in the outer mitochondrial membrane, releasing cytochrome c from the mitochondria, causing the ATP-dependent formation of the apoptosome, a large multiunit complex of APAF-1 and caspase-9. The apoptosome provides a platform for caspase-9 activation, and the activated protease then cleaves executioner caspases, caspase-3 and -7, which will enact irreversible cell death. Extrinsic apoptosis is a response to extracellular pro-death signals. Three major classes of pro-death receptor-ligand pairs have been well described: Fas and Fas ligand, TNFα and the TNF receptor, and the death receptors DR4 and DR5 and TRAIL (Xu and Shi, 2007). Activation of these pathways triggers the formation of the death-induced signaling complex (DISC), to act as a platform for activation of caspase-8 or -10, which can then go on to cleave and activate effector caspases. In hemorrhagic stroke tissue, this extrinsic pathway is likely triggered by intracellular contents from other dying cells.

Apoptosis is observed as a large portion of cell death in hemorrhagic stroke. A study in 2003 by Qureshi et al. showed that in the perihematomal region in clinical cases of intracerebral hemorrhage, a majority of the cells were positive for terminal deoxynucleotidyl transferase-mediated 5-triphosphate nick-end (TUNEL) labeling (Qureshi et al., 2003). This study likely represents a plurality of modes of cell death, rather than just apoptosis, as the authors initially claimed, as TUNEL-positivity can be indicative of more than apoptosis (Kelly et al., 2003; Grasl-Kraupp et al., 1995). Another study has found that this proportion of apoptotic cells increases to a peak in the first 12–24 h in the perihematomal tissue after intracerebral hemorrhage, measured as an increase in TUNEL positivity and Bax protein and mRNA expression (Guo et al., 2006). This indicates that a large portion of cells that are near the most affected areas are undergoing apoptotic death, presumably stimulated by pro-death signals received from the brain parenchyma. Additionally, in a dog model of subarachnoid hemorrhage, caspase inhibitors were able to protect against some of the behavioral effects, indicating that caspase-3 activity is an important pathological mechanism of hemorrhage (Zhou et al., 2004). Altogether, apoptosis is likely to be present in both subarachnoid and intracerebral hemorrhage tissue, and blocking apoptosis may be a plausible therapeutic route.

## 3 Necrosis

Necrosis is a form of uncontrolled, pro-inflammatory cell death that occurs under damaging conditions. Necrosis is characterized pathologically by membrane breakdown and cytoplasmic swelling, without the chromatin condensation that is characteristic of apoptosis (Khalid and Azimpouran, 2025). Necrosis is triggered by cell-external factors, such as mechanical breakdown of tissue or intense ischemia. Necrotic cells release intracellular contents, which act as pro-inflammatory damage-associated molecular patterns (DAMPs), triggering an immune response. Necrosis is understood as an unregulated, energy-independent, and non-cell-initiated form of death, but there are ways that a cell can induce its own pro-inflammatory necrotic fate through necroptosis or pyroptosis, as described later in this paper.

Necrosis is responsible for a large amount of cell death during the primary and short-term secondary injury after a hemorrhagic stroke. The mechanical force generated from increased intracranial pressure causes cells to rupture and become necrotic. It has been shown in samples taken from patients with intracerebral hemorrhage that around 25% of cells in the perihematomal region are necrotic at 24 h post-stroke (Qureshi et al., 2003). Since necrosis is a physical consequence of traumatic environmental changes that occur during the primary injury during a hemorrhagic stroke, modification with therapies presents a significant challenge. There are interventions that can block oxidative stress-induced death by scavenging reactive oxygen species, which have shown some beneficial preclinical effects in ischemic injury, but have not been tested for application in hemorrhagic stroke (Hwang et al., 2018).

## 4 Necroptosis

Necroptosis is an induced form of necrosis. This is a pro-inflammatory death in response to either an external factor, such as ligands for Toll-like receptors, including the TNFα receptor, or an abundance of intracellular reactive oxygen species (ROS) (Dhuriya and Sharma, 2018).

A cell can induce its own necrotic death in response to extrinsic factors secreted by the cell, most importantly in response to the stimulation of its death receptors, including by tumor necrosis factor 1 (TNFα). TNFα binds to the TNFα receptor in the plasma membrane, causing a conformation shift in the receptor’s luminal domain, which starts the platform for the membrane-associated Complex I. Complex I is made up of TRADD, FADD, RIPK1, TRAF2/TRAF5, and cIAP1/cIAP2, and causes a variety of post-translational modifications of TRADD, TFNR1, and RIP1, including the ubiquitination of RIP1. A portion of Complex I containing RIP1, TRADD, FADD, and TRAF2 then detaches from the membrane, where it forms Complex II by incorporating caspase-8 and RIPK3. Activated RIPK3 can then phosphorylate the pseudokinase MLKL, which enacts necroptosis through permeabilization of various intracellular membranes (Martens et al., 2021). Thus, phosphorylated MLKL is a *bona fide* marker for necroptotic cells.

Whether this cell proceeds to a necroptotic fate, or apoptosis is triggered, is dependent on caspase-8 (and caspase-10 in humans). If procaspase-8 is present in the cell when RIPK1 is released from the membrane, caspase-8 will form a complex with it and FADD and cleave itself. This activated form of caspase-8 will then enact apoptosis, suppressing necroptosis. This then positions the presence of caspase-8 as the switch between a cell’s necroptotic and apoptotic fates. Since caspase-8 is differentially expressed in different cell populations, whether they face apoptosis or necroptosis is partially determined by the presence of caspase-8 (Velier et al., 1999). For example, different subpopulations of pyramidal neurons differentially express capsase-8, and that affects their longevity in a rodent model of ischemic stroke.

Necroptotic death has been shown to be an abundant form of death in response to blood and blood oxidation products, such as heme. A dog model of subarachnoid hemorrhage has shown that TNFR1 is present in dying cells after injury (Zhou et al., 2004), indicating that the signaling mechanism to initiate necroptosis is present. Additionally, Necrostatin-1, an inhibitor of RIPK1, has been shown to reduce neurovascular injury after collagenase-induced hemorrhage in rodents (Chang et al., 2014). Taken together, these findings demonstrate that necroptosis may be contributing to the pathology of hemorrhagic stroke in this model, and presents the necroptotic pathway as a potential therapeutic target. This implicates necroptosis as a key pathological mechanism of cell death in hemorrhagic stroke.

## 5 Pyroptosis

Pyroptosis is another pro-inflammatory method of cell death, characterized by the release of IL-1β and IL-18 from the dying cell. When DAMPs bind to their conjugate receptors, including NLRP3, DNA receptors, and the pyrin receptor, they trigger the formation of the inflammasome, a large protein complex, which serves as an activating platform for caspase-1 (Bertheloot et al., 2021). Caspase-1 cleaves pro-IL-1β and pro-IL-18 into their mature forms (IL-1β and IL-18 respectively), as well as cleaving Gasdermin D (GSDMD) into a form that can form pores in the plasma membrane. This breakdown of the plasma membrane causes the death of the cell, as well as the release of the newly synthesized pro-inflammatory cytokines. This triggers an immune response through the direct sensing of these cytokines by immune cells.

The activity of caspase-1, an essential step for pyroptosis, has been shown to be required for the breakdown of the BBB during collagenase-induced hemorrhage in mice (Lin et al., 2018). Indeed, an inhibitor of caspase-1, AC-YVAD-CMK, has been shown to inhibit pyroptosis and protect against motor deficits in this model. However, it is important to know that this model of stroke likely overrepresents immune activation, as collagenase itself causes immune cell activation, independent of its effect on the BBB (Singh and Bhattacharyya, 2014).

In perihematomal tissue from patients with ICH, single cell sequencing analysis has shown that a transcriptionally unique population of IL-1β^+^ microglia undergoes pyroptosis within the first 2 days after injury (Zhang et al., 2024). These pro-inflammatory dying microglia exist in a balance with another population of microglia, identified as expressing P2RY12. As a therapeutic target, this molecular distinction of at least two microglial subpopulations is important, since strategies manipulating only pro-inflammatory cells could be beneficial (Scott et al., 2021).

## 6 Ferroptosis

Ferroptosis is an iron-dependent method of cell death (Dixon et al., 2012). In intracerebral hemorrhage, lysed red blood cells release hemoglobin into the neural parenchyma. The iron bound to this hemoglobin leads to the generation of reactive oxygen species, in a mitochondria-dependent manner. An increase in the local concentration of iron also disrupts normal iron metabolism (Koduri et al., 2022). Ferroptosis exists as a unique death pathway as it does not use energy and does not involve caspases or lysosomes (Dixon et al., 2012; Wang et al., 2002; Zille et al., 2017).

Ferroptosis has been shown to be active in oligodendrocyte precursor cells in a mouse model of intraventricular hemorrhage (Shen et al., 2022). Additionally, it has been confirmed that inhibition of ferroptosis with ferrostatin-1 protects against neuronal death in the collagenase model of intracerebral hemorrhage in mice (Zille et al., 2017; Li et al., 2017). This effect may be through the rescue of cells that would otherwise die due to iron overload, or it could be through an indirect, synergistic effect on inflammation, as ferroptosis has been shown to be immunogenic (Zhang et al., 2018).

## 7 Autophagic cell death

Autophagy is a common mechanism for cell death and clearance after damage. Autophagy is triggered by activation of the mTOR pathway (Zhang et al., 2022). When an organelle is recognized for autophagy, a second membrane is formed around the organellar membrane, and that then fuses with the lysosome, acidifying the autophagosome (Towers and Thorburn, 2016). This begins the breakdown of the vesicle contents. While autophagic materials are usually used for the clearance of defective organelles or cytoplasmic components, excessive autophagy can cause cell death via a pathway that is distinct from apoptosis or necroptosis (Denton and Kumar, 2019).

Autophagy and phagocytosis work through similar mechanisms, and are thus inextricably linked. There is evidence that increased autophagy can modulate microglial receptiveness to inflammatory signaling, and defective autophagy may impair phagocytic function (Chen et al., 2022). Thus, microglial autophagy is an essential player in the secondary injury from hemorrhagic stroke.

The presence of autophagic cell death has been verified in a rat model of subarachnoid hemorrhage by electron microscopic identification of autophagosomes and autolysosomes (Lee et al., 2009). Additionally, Beclin-1, a marker of autophagic tissue, has been observed in a collagenase mouse model of intracerebral hemorrhage (Shen et al., 2016). This same study also found that there is an increase in NF-κβ signaling that can be reduced by blocking autophagy, indicating that this pathway contributes to inflammation and immune response. Inhibition of autophagy has also been shown to be beneficial by preventing ferroptotic brain injury (Chen et al., 2012). While autophagy is essential for clearance of debris by macrophages, this indicates that autophagy may also be playing a deleterious role in hemorrhagic stroke pathology, and therefore could serve as a therapeutic target, with careful consideration of its beneficial role. Currently, therapeutics targeting autophagy are being investigated in many diseases, including neurodegenerative and infectious (Towers and Thorburn, 2016).

## 8 Therapeutic considerations and conclusion

Under the conditions of hemorrhagic stroke, it is likely that all these death pathways are occurring at some level. By understanding the contribution and impact that each of these distinct molecular death pathways has on the pathology of hemorrhagic stroke, it may be possible to design specific therapeutics that can target only the negative consequences of hemorrhagic stroke.

Investigations into inhibitors of cell death pathways have provided promising preclinical results but have not yet translated into successful clinical trials. In a screen of many inhibitors of different cell death pathways, inhibitors of ferroptosis demonstrated the most consistent protection against hemin toxicity in cultured cortical neurons (Zille et al., 2017). Ischemia, however, has been shown *in vivo* to cause sequential waves of necroptosis and apoptosis (Naito et al., 2020). Therefore, therapeutics that can address a single pathology of hemorrhagic stroke may not be able to ameliorate the effects of a stroke, due to the multiplicity of cell death pathways that have been shown to be active. Uncovering interplay between these pathways will also be essential for developing effective treatments.

To effectively evaluate the efficacy of therapeutics, there must be testing in a multimodal and human-relevant disease model (Table 1). Subarachnoid hemorrhage can be effectively replicated in rodents through endovascular perforation, in which blood vessels are ruptured from the inside (Schüller et al., 2013). Intracerebral hemorrhage is typically either modeled through intracerebral injection of autologous blood, to recreate hemotoxic effects, or collagenase, which degrades extracellular matrix including the tight junctions that hold together blood vessels (Thangameeran et al., 2022; MacLellan et al., 2010). Both models are able to model some portion of intracerebral hemorrhage, but neither fully recapitulate the vasogenic nature of the injury. To understand how different methods of cell death interact in a disease state, a better animal model of intracerebral hemorrhage is critical.

**TABLE 1:** Common animal models of hemorrhagic stroke.

Model	Description	Citations
Endovascular perforation	A blood vessel is surgically punctured with a sharp filament or microelectrode. This effectively models the vascular blood in neural parenchyma	Liu et al. (2021), Barry et al. (1979)
Collagenase injection	Bacterial collagenase is injected, breaking down extracellular matrix and tight junctions between endothelial cells. Importantly, bacterial collagenase exacerbates the inflammatory response, and so this model is most effective for studying non-inflammatory pathology	Rosenberg et al. (1990), Krafft et al. (2012)
Autologous blood injection	Blood is injected to the target site. Can model either subarachnoid or intracerebral hemorrhage, based on injection site. Effectively models toxicity of blood, and can be used to model hematoma expansion to enable comparison to progression in humans	Krafft et al. (2012), Rynkowski et al. (2008)

Additionally, stem cell transplantation has also been investigated, based on the findings that the certain populations of stem cell can provide an anti-inflammatory and pro-growth environment through secreted factors (Stonesifer et al., 2017). As mentioned earlier, investigations in a mouse model of intraventricular hemorrhage has shown that oligodendrocyte precursor cells die by ferroptosis, thus the protection or replacement of these cells may protect white matter connections in brain tissue (Shen et al., 2022).

Ultimately, hemorrhagic stroke is a disease with a complex pathology which involves multiple mechanisms for cell death. With continued research, we can develop a better understanding of disease progression, and we may be able to develop treatments that can be powerful strategies to prevent brain cell death in hemorrhagic stroke.
